# Experimental and computational analysis of SnS_x_ encapsulated into carbonized chitosan as electrode material for potassium ion batteries

**DOI:** 10.1038/s41598-024-82588-0

**Published:** 2024-12-28

**Authors:** Andrzej P. Nowak, Anna Rokicińska, Zhilong Wang, Marta Prześniak-Welenc, Zuzanna Zarach, Kehao Tao, Daria Roda, Mariusz Szkoda, Konrad Trzciński, Jinjin Li, Piotr Kuśtrowski

**Affiliations:** 1 Faculty of Chemistry, Gdańsk University of Technology, Narutowicza 11/12, 80-233 Gdańsk, Poland; 2 Advanced Materials Center, Gdańsk University of Technology, Narutowicza 11/12, 80-233 Gdańsk, Poland; 3 Faculty of Chemistry, Jagiellonian University, Gronostajowa 2, 30-387 Kraków, Poland; 4 National Key Laboratory of Advanced Micro and Nano Manufacture Technology, Shanghai Jiao Tong University, Shanghai, 200240 China; 5 Department of Micro/Nano Electronics, School of Electronic Information and Electrical Engineering, Shanghai Jiao Tong University, Shanghai, 200240 China; 6 Institute of Nanotechnology and Materials Engineering, Gdańsk University of Technology, Narutowicza 11/12, 80-233 Gdańsk, Poland

**Keywords:** Potassium-ion battery, Chitosan, Tin sulphide, Electrode material, Computational modeling, Chemistry, Energy science and technology, Engineering, Materials science, Mathematics and computing

## Abstract

Tin sulphide compounds (SnS_x_, x = 1, 2) are potential anode materials for potassium-ion batteries (PIBs) due to their characteristic layered structure, high theoretical capacity, non-toxicity and low production cost. However, they suffer from significant volume changes resulting in poor performance of such anodes. In this work incorporation of SnS_x_ into the carbon structure was expected to overcome these disadvantages. Two SnS-based electrode materials encapsulated into chitosan, as a natural carbon source, are fabricated by two different synthesis routes: (a) solvothermal, and (b) solvothermal followed by pyrolysis. The results indicate that the synthesis route is a crucial factor affecting the composition and electrochemical performance of the negative electrode. The electrode material, exhibiting a high reversible capacity (304 mAh/g at 50 mA/g), and good rate capability (128 mAh/g at 1000 mA/g for 500 cycles) is produced by the solvothermal method. The relationship between specific capacity and synthesis procedure is analyzed using the results obtained from XRD, XPS. Additionally, density functional theory is employed to provide deeper insights into the underlying mechanisms governing the electrochemical performance of the SnS_x_@C electrode materials.

## Introduction

Lithium-ion batteries (LIBs) are the most common energy storage sources utilized worldwide^1^. They can offer relatively high energy density, light design, long lifespan and low environmental impact in comparison with other battery systems such as nickel–cadmium (NiCd), and nickel-metal hydride (NiMH). Moreover, the ongoing miniaturization of electric and electronic equipment resulted in the small, mobile electronic equipment becoming an indispensable attribute of the modern human. This leads to an increase in demand for LIBs energy storage systems as well as it is particularly visible in increasingly popular hybrid cars, plug-in hybrid cars or electric cars, prices of which significantly decreased recently. For that reason, much attention is being paid to research on the new generation of high-energy lithium batteries, the operating parameters of which would make powering zero-emission electric motors possible^2^. However, the rarity in the earth’s crust of lithium (0.0017 wt%) and the increasing cost of lithium compounds have raised concerns over the long-term and large-scale supply availability of LIBs. Therefore, great efforts have been devoted to exploring new rechargeable battery alternatives to LIBs, i.e., sodium-ion batteries (SIBs)^3^ or potassium-ion batteries (PIBs)^4^. PIBs are an area of great interest as potential candidate for LIBs replacement. It is due to the fact that potassium has advantages over lithium ions, such as abundant terrestrial resources (2.09 wt%), low cost, and redox potential close to that of Li^+^ /Li (+ 0.1 V vs. Li^+^/Li). Additionally, potassium exhibits a lower reduction potential than Li^+^/Li in non-aqueous solvents i.e., propylene carbonate (− 0.09 V vs. Li^+^/Li), ethylene carbonate/diethyl carbonate (− 0.15 V vs. Li^+^/Li)^5^, which makes PIBs more attractive than LIBs in terms of operating at higher potentials with considerable energy density. Therefore, PIBs can become powerful competitors to LIBs in the near future, especially for large-scale applications for energy storage and conversion.

All components of PIBs i.e. cathode material, anode material, electrolyte and separator play a vital role in the electrochemical performance of PIBs^6^. In the case of anode materials, K-metal is a priority due to its high theoretical capacity and high energy density. However, it suffers from dendrite growth as a result of uneven electron distribution during reduction^7^. Thus, an appropriate anode material is required for practical application. Depending on the charge storage mechanism, there are three classes of materials utilized as anodes for PIBs: (1) alloying materials, (2) intercalation materials and (3) conversion materials^8^. Among them, the conversion and alloy materials attract considerable attention due to their high theoretical capacity, and redox reversibility in comparison with intercalation materials. The general equations for conversion reaction (1) and alloying (2) are given below:

1 $${\text{A}}_{{\text{x}}} {\text{B}}_{{\text{y}}} + \left( {y \cdot n} \right){\text{M}} + = x{\text{A}} + y{\text{M}}_{{\text{n}}} {\text{B}}$$

2 $$x{\text{A}} + y{\text{M}}^{ + } + y{\text{e}}^{-} \to {\text{A}}_{{\text{x}}} {\text{M}}_{{\text{y}}}$$

where A is a transition metal, B is an anion, M is an alkali metal, and *x*, *y* are stoichiometric numbers.

One of the most promising negative electrode materials for PIBs is a group of transition metal sulfides^9^, among which, tin sulfide is gaining special recognition. It may exist in two stable forms: SnS and SnS_2_, with the theoretical capacity of the former of 1136 mAh/g and the latter of 733 mAh/g. The large lattice spacing for both SnS_2_ (0.59 nm)^10^ and SnS (0.39 nm)^11^, compared with Stokes radius of K^+^ in organic solvents (0.36 nm)^12^, evidence that the unique layered structure of SnS_x_ enables potassium ions to be inserted/extracted into/from interlayers. The electrochemical reaction mechanism between SnS_x_ and K^+^ may be presented as follows^13^:

3 $${\text{SnS}}_{{\text{x}}} + {2}x{\text{K}}^{ + } + {2}x{\text{e}}^{ - } \to {\text{Sn}} + x{\text{K}}_{{2}} {\text{S}}$$

4 $${\text{Sn}} + y{\text{K}}^{ + } + y{\text{e}}^{ - } \to {\text{K}}_{{\text{y}}} {\text{Sn}}$$

However, enormous volume changes during charge/discharge processes lead to poor electrochemical performance i.e. cycling stability, electronic conductivity, and reversible capacity. To improve the electrochemical activity of SnS_x_, an approach of introducing various carbon materials into the structure was presented several times in the literature^14,15^, with the assumption that carbon matrix plays a crucial role in the accommodation of volume changes, enhancing electronic conductivity of tin sulfide, and finally limiting the dissolution of the polysulfide intermediates formed during the insertion process^16^.

Here, we show SnS_x_ encapsulated into a carbon matrix, which was formed from chitosan as a natural carbon precursor. Our recent studies evidenced that pyrolyzed chitosan can be utilized as a stress-accommodating phase in anode manufacturing^17^. In the present study, we used two routes for the electrode material’s synthesis: (1) solvothermal reaction, and (2) solvothermal reaction followed by pyrolysis. These two methodologies were selected to investigate how variations in synthesis methods could influence the electrochemical performance of the resulting electrode materials.

The obtained hybrid materials have been investigated using solid-state physics techniques such as X-ray diffraction (XRD), X-ray photoelectron spectroscopy (XPS), Raman spectroscopy, scanning electron microscopy (SEM) coupled with energy dispersive X-ray spectroscopy (EDX), electrochemical polarization methods and DFT calculations.

## Experimental

### Synthesis of SnS_x_@C

Anhydrous SnCl_4_ (3.1 g) (POCH, Gliwice, Poland), chitosan (2.36 g) (MMW, 75–85% deacetylated, Sigma Aldrich, St. Louis, MO, USA), and thioacetamide (0.96 g) (Chemat, Gdańsk, Poland) were combined with ethylene glycol (100 ml) (POCH, Gliwice, Poland). The mixture was then placed in a Teflon-lined autoclave and heated at 150 °C for 20 h. This resulting material is referred to as SnS_x_@C_s. After cooling to room temperature, the resulting powder was collected and washed multiple times with acetone. A portion of the product underwent pyrolysis at 600 °C for 6 h in an Ar atmosphere, resulting in the material SnS_x_@C_sp. Finally, the product was dried under vacuum at 110 °C for 24 h.

### Material characterization

The phase composition of the electrode materials was determined using X-ray diffraction (XRD) within a 2θ range of 10°–70°, utilizing a Philips Xpert PRO-MPD diffractometer with copper K_α_ radiation (λ = 1.5404 Å). The surface composition was analyzed through X-ray photoelectron spectroscopy (XPS). Spectra were acquired on a Prevac spectrometer equipped with a VG SCIENTA R3000 hemispherical analyzer (pass energy of 100 eV) and a monochromated AlK_α_ source (1486.6 eV). Binding energies were calibrated to the C 1*s* peak at 284.8 eV. After subtracting the Shirley background, the raw spectra were fitted with Gaussian–Lorentzian shapes using Casa XPS software.

The surface morphology of the samples was examined using a Thermo Fisher Scientific Apreo 2S LoVac scanning electron microscope (Waltham, MA, USA) equipped with an energy dispersive X-ray spectroscopy (EDX) detector. Imaging was performed in backscattered electron (BSE) mode with a 20 kV accelerating voltage and a beam current of 0.40 nA.

### Electrode preparation

The electrodes were prepared by creating a slurry composed of an 8:1:1 weight ratio of SnS_x_@C, a binder (polyvinylidene fluoride, Solef® 6020, Solvay, Belgium), and carbon black (Super P®, Timcal Ltd., Switzerland) in N-methyl-2-pyrrolidinone (NMP) solvent (AlfaAesar, USA). This slurry was then applied onto a metallic current collector made of copper foil (Schlenk Metallfolien GmbH & Co KG, Germany) and allowed to dry at room temperature for 24 h. Afterwards, 10 mm diameter discs were punched out and further dried at 110 °C for 24 h under a dynamic vacuum in a Glass Oven B-595 (Büchi, Germany). The electrode material’s mass loading was approximately 3 mg/cm^2^.

### Electrochemical measurements

The electrochemical tests were conducted in a two-electrode pouch cell, with potassium metal serving as both the counter and reference electrode and the material under investigation as the working electrode. The electrolyte used was 0.8 M KPF_6_ (Merck, Germany) in a 1:1 weight ratio of EC:DMC (both Sigma-Aldrich, Germany), while glass fiber (Schleicher & Schüll, Germany) was employed as the separator. A galvanostat/potentiostat (ATLAS 1361 MPG&T, Gdańsk, Poland) was used within a potential range of 0.01–3 V versus K/K^+^ or 0.01–2.0 V to study the charge storage mechanism via cyclic voltammetry and chronopotentiometry. The galvanostatic intermittent titration technique (GITT) was performed with a current pulse of 25 mA/g for 30 min, followed by a 2-h open circuit voltage period, on electrode materials after the first charge/discharge cycle. Electrochemical impedance spectroscopy (EIS) measurements were carried out using a potentiostat with an integrated impedance analyzer (CompactStat, Ivium Technologies, Eindhoven, The Netherlands) over a frequency range of 100 kHz to 10 mHz with a 10 mV amplitude for electrode material before charging.

## Results and discussion

### Solid-state physics characterization

#### SEM and EDX

**Fig. 1:**
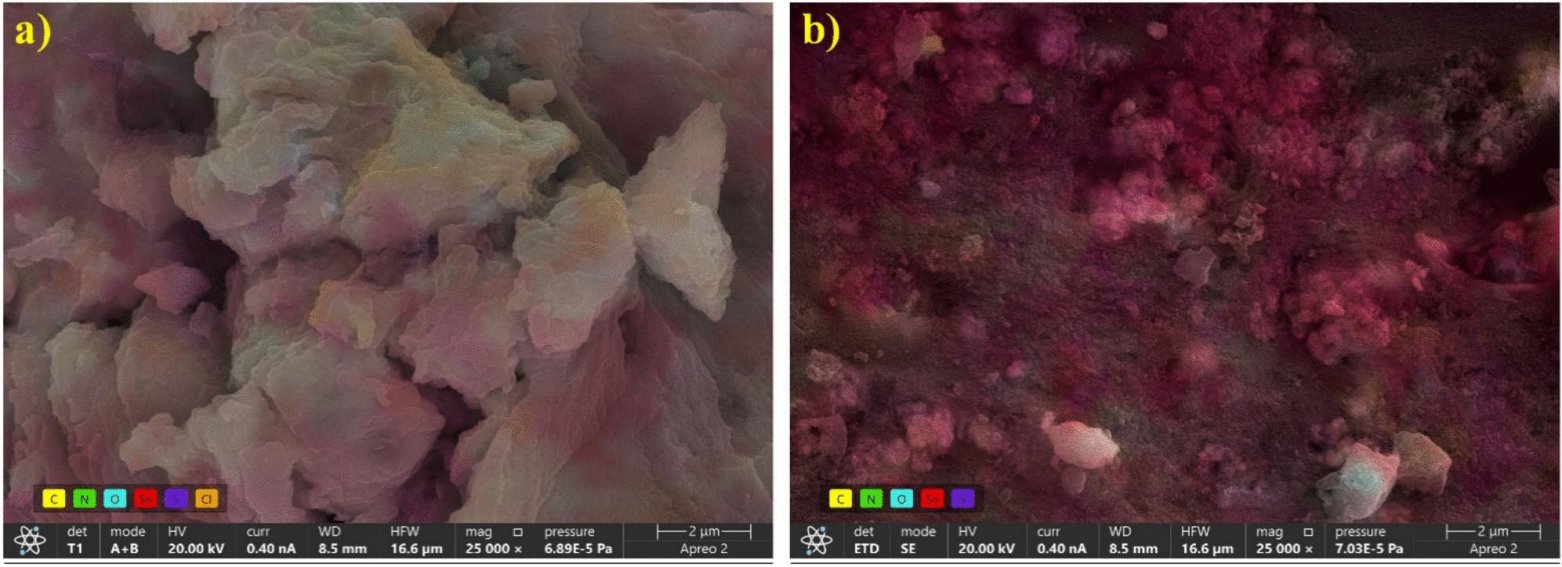
SEM images with EDX mapping of SnS_x_@C_s (**a**) and SnS_x_@C_sp (**b**) materials.

Figure 1 shows the distribution of elements of the synthesized materials determined by SEM imaging coupled with elemental mapping (EDX). Based on the SEM images, we observe that the SnS_x_@C_s material consists of larger crystallites compared to SnS_x_@C_sp. This suggests that the pyrolysis process results in a smaller crystalline structure. The difference in crystallite size can significantly influence the material’s properties, including its surface area, porosity, and potential applications in energy storage.

The common feature of the images is the confirmation of the presence of Sn, S, O, N and C in the samples. The S/Sn atomic ratio is 1.9:1 and 1.4:1 for SnS_x_@C_s and SnS_x_@C_sp, respectively. It evidences the formation of SnS_2_ in the former and the mixture of SnS_2_/SnS in the latter. Detailed information on the material composition based on the EDX studies is given in the supplementary information (Figs. S1 and S2).

#### XRD

**Fig. 2:**
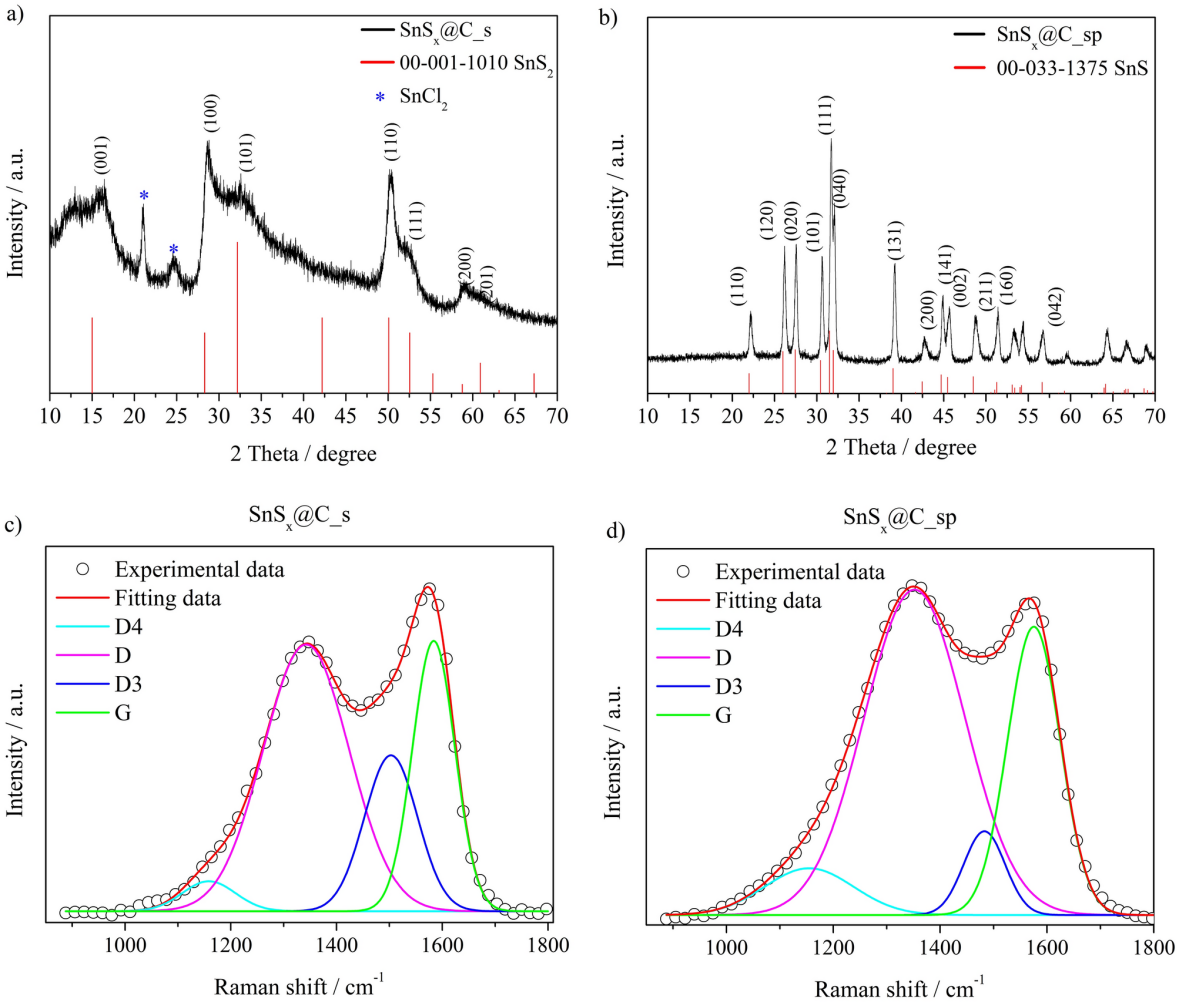
XRD patterns and Raman spectra of SnS_x_@C_s (**a**, **c**) and SnS_x_@C_sp (**b**, **d**) materials.

Figure 2a and b show the XRD patterns of SnS_x_@C_s and SnS_x_@C_sp, respectively. The diffractogram of the former sample indicates main peaks at approximately 15°, 28°, 32.5°, 50.5°, 52.7° and 60°, corresponding to the tin(IV) sulfide (JCPDS card no. 00-001-1010), with a hexagonal crystal structure. The relatively broad reflections suggest the SnS_x_ low crystallinity degree and small grain size according to Scherrer’s equation. Additionally, the diffraction peaks observed at approximately 20.5° and 24.5° are attributed to the presence of tin chloride SnCl_2_ (JCPDS card no. 01-086-1131) formed during the solvothermal process. The presence of SnCl_2_ suggests that the solvothermal treatment of SnCl_4_ in the presence of thioacetamide led to the chemical reaction resulting in the synthesis of SnS_2_ as well as the reduction of SnCl_4_ to SnCl_2_. Moreover, the Cl^-^ ions disappeared after the pyrolysis of the sample (see, Table S2).

In the diffractogram of the sample after pyrolysis, SnS_x_@C_sp in Fig. 2b, the observed peaks can be confidently indexed within the orthorhombic system SnS phase (JCPDS card no. 00-033-1375). Notably, no other peaks were detected, confirming the synthesis of SnS without any impurities. At elevated temperature, and in the presence of carbon phase, tin (IV) is partially reduced to tin (II), as was confirmed by EDS analysis. The usage of the carbon phase in carbothermal reduction of SnS_2_ to SnS was already described by Song et al.^18^ Thus, it seems that the change of the crystal structure of SnS_2_ from hexagonal to amorphous is needed for the carbothermal reduction process. This may be also a reason why we did not observe the ordered crystal structure of SnS_2_ in the sample after pyrolysis (SnS_x_@C_sp).

#### Raman spectroscopy

To investigate the influence of the carbon phase on the electrochemical performance of electrode materials Raman spectroscopy was applied. The Raman spectra of SnS_x_@C_s and SnS_x_@C_Sp are shown in Fig. 2c and d, respectively. The shape of both spectra is similar, however, in the case of the SnS_x_@C_s material, the peak at ~ 1600 cm^−1^ is more intense than the peak at ~ 1350 cm^−1^, which is not observed for the latter electrode. Nevertheless, it is known that the proper analysis of Raman spectra of the carbon material in the range from 1000 to 1800 cm^−1^ requires taking into account the presence of both crystalline and amorphous carbon domains. Thus, we performed curve fitting assuming Gaussian band shapes. A Gaussian peak is known to be more accurate for a random distribution of phonon lifetimes in disordered materials^19^. Four maxima at ~ 1200 cm^−1^, ~ 1350 cm^−1^, ~ 1500 cm^−1^, and ~ 1580 cm^−1^ were identified. The first three are attributed to the presence of disordered graphite-like species (D bands), and the last one confirms the presence of in-plane bond stretching of sp^2^-ring carbon atoms of graphitic lattice (G band)^20^. The relative intensities of D and G bands are affected by the type of carbon material^21^. The analysis allows the calculation of particle size of the crystalline carbon domains, known as cluster size (*L*_a_), from Eq. (5) proposed by Ferrari and Roberts for disordered and amorphous carbons^19^:

5 $$\frac{I\left( D \right)}{{I\left( G \right)}} = 0.0055 \cdot L_{a}^{2}$$

The calculated cluster size is equal to 1.34 nm and 1.43 nm for the SnS_x_@C_s and the SnS_x_@C_sp, respectively. This is much lower than the grain size of graphite and similar to the case of pyrolysed lignin^22^. The values of the calculated *L*_a_ parameter for the SnS_x_@C_s and SnS_x_@C_sp are similar, and thus it may suggest that the presence of the carbon phase does not significantly affect the electrochemical performance of the studied electrode materials, see Fig. 4.

#### XPS

**Fig. 3:**
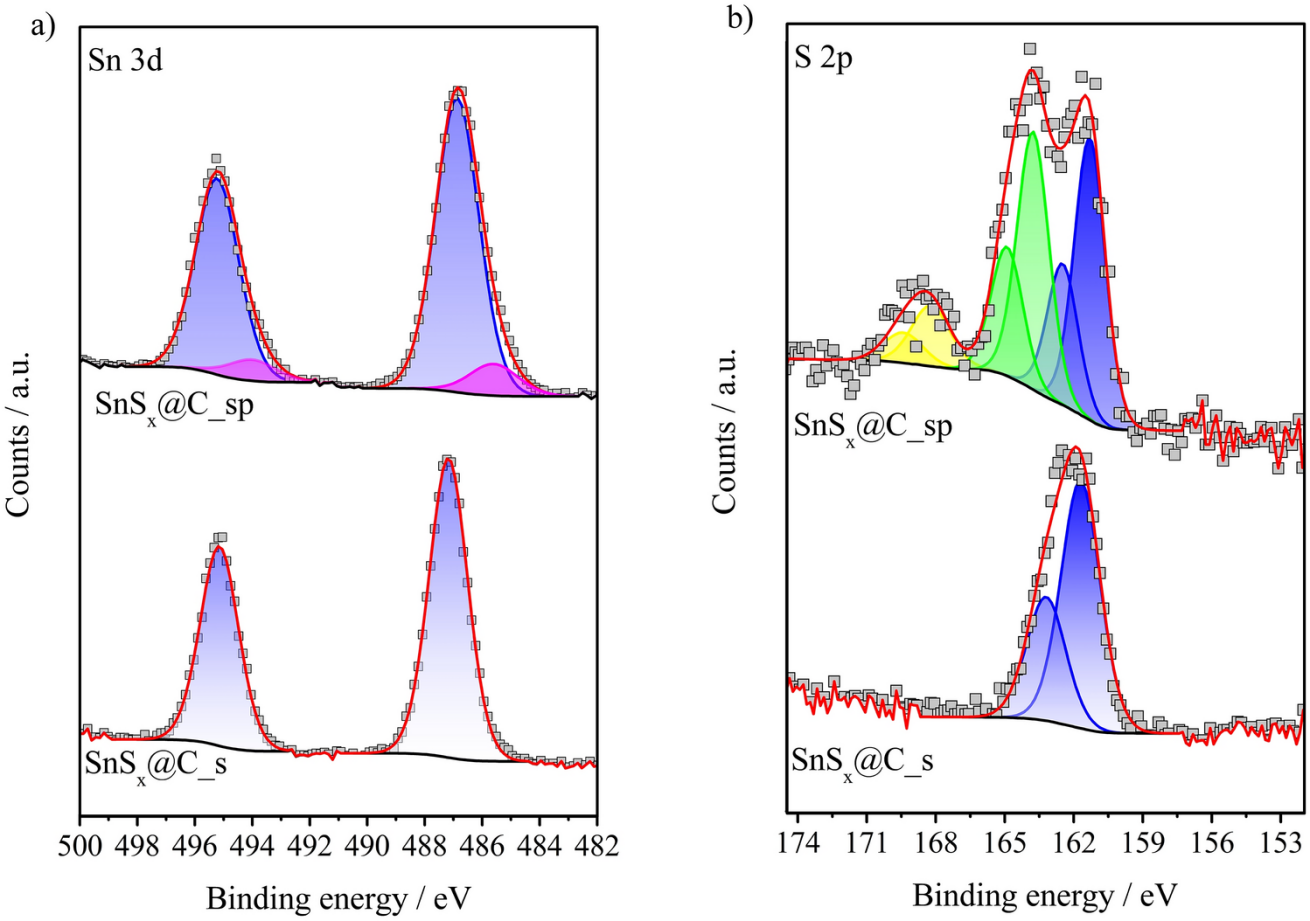
High resolution XPS spectra of (**a**) Sn 3d and (**b**) S 2p for the SnS_x_@C_s and SnS_x_@C_sp electrode materials.

The XPS Sn 3d and S 2p spectra (Fig. 3) clearly confirm that the SnS_x_@C_s material is dominated by the presence of Sn^4+^ bound to sulfide ions (S^2−^). This is evidenced by the position of the Sn 3d_5/2_ and Sn 3d_3/2_ peaks at binding energies of 486.5 eV and 494.9 eV, respectively, as well as the doublet of S 2p_3/2_ (161.7 eV) and S 2p_1/2_ (163.2 eV)^23,24^. It is worth noting that the surface S/Sn atomic ratio of 1.7 (determined based on the cumulative peak areas of S 2p and Sn 3d) indicates a partial balancing charge of Sn^4+^ by other surrounded anions, such as O^2−^, OH^−^ or Cl^−^. After pyrolysis, when the organic components were decomposed and transformed into carbon species, in addition to Sn^4+^, the presence of Sn^2+^ (approximately 10% of the total surface Sn content) is also identified on the surface of the tested sample through the Sn 3d_5/2_ and Sn 3d_3/2_ peaks located at 485.6 eV and 494.0 eV, respectively^23^. Furthermore, the thermal treatment significantly changed the form of surface sulfur. In the XPS S 2p spectrum, the peaks at 162.5 eV (S 2p_3/2_) and 164.9 eV (S 2p_1/2_), attributed to disulfides, and at 168.2 eV (S 2p_3/2_) and 169.4 eV (S 2p_1/2_), corresponding to sulfates, are distinguished^25^. The roughly determined distribution of S species shows content of 44% S^2−^, 43% S_2_^2−^ and 12% SO_4_^2−^. The surface S/Sn atomic ratio for this sample is 0.9. This means that a large part of S is released during pyrolysis. On the other hand, the resulting highly defective surface is easily oxidized by atmospheric air, which is in agreement with the XRD data. The presence of tin(IV) sulfide in SnS_x_@C_sp, see Fig. 2a, was not confirmed by XRD. It may be assumed that the pyrolysis of the material resulted in the disappearance of the crystalline structure of SnS_2_, similar to what was previously observed with exfoliated WO_3_^26^. On the other hand, the measured XPS C 1s and O 1s spectra (Fig. S3) confirm the successful transformation of the chitosan structure present in the SnS_x_@C_s material to the form of carbon in the sample after carbonization^27,28^.

### Electrochemistry

**Fig. 4:**
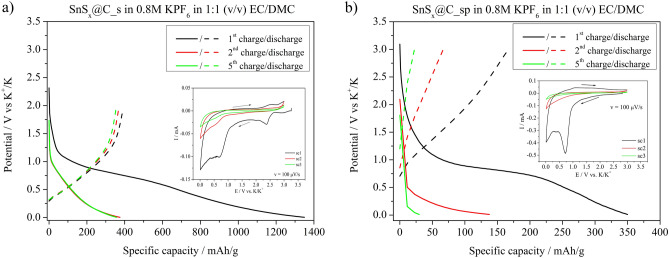
Galvanostatic charge/discharge curves recorded for the (**a**) SnS_x_@C_s, and (**b**) SnS_x_@C_sp electrode materials at *j* = 50 mA/g in the potential range from 0.005 to 3.0 vs. K/K^+^.

The galvanostatic charging/discharging curves of the SnS_x_@C_s and SnS_x_@C_sp electrode materials, recorded at *j* = 50 mA/g, are shown in Fig. 4a and b, respectively. There is a broad plateau starting at ~ 1.0 V in the first reduction reaction (charging). This plateau is also visible on the cyclic voltammetry curve (CV) (see inset in Fig. 4) during the first charging. Several processes may occur during primary K^+^ insertion into the SnS_x_@C electrode material, including K^+^ intercalation into the carbon matrix, followed by solid electrolyte interphase (SEI) formation; Sn formation according to Eq. 3; and tin-potassium alloy formation (Eq. 4). Taking into account that the plateau is observed only in the first cycle, it is believed that it is attributed to the irreversible decomposition of SnS_x_ coupled with SEI layer formation. For the SnS_x_@C_s, the specific charge capacity of the first cycle was 1349 mAh/g, while for the discharge the specific capacity value was 394 mAh/g. It gives an immense irreversible capacity loss (ICL) in the first cycle equal to 955 mAh/g. In the next cycles, the ICL was 3 mAh/g and 1 mAh/g, respectively. In the case of SnS_x_@C_sp, the specific capacities were much lower showing 350 mAh/g for the first charge, and 167 mAh/g for the first discharge, and still the specific capacity of subsequent cycles exhibited noticeable capacity loss. This phenomenon evidences that the synthesis procedure is a crucial factor influencing the electrochemical properties of electrode material due to differences in the composition between SnS_x_@C_s and SnS_x_@C_sp. It shows that the presence of the carbon phase does not play a significant role in the electrochemical performance of electrode material, as we observed in our previous studies^29^. However, the presence of carbon matrix and SEI formation affect the capacity of electrode material in the first cycle. The high reversibility of the electrochemical reaction of SnS_x_@C_s in comparison with SnS@C_sp in the second and third charge/discharge cycles may indicate superior stability during the cycling of the former one. It is worth mentioning that clear cathodic or anodic current maxima were not observed on cyclic voltammetry curves for both electrode materials. It evidences that there is no simple conversion nor alloying mechanism as given in^13^. However, the shape of cv curves for SnS_x_@C_s and SnS_x_@C_sp differs in the potential range from 0.005 to 1.0 V. In the case of SnS_x_@C_s electrode material, one may see a peak-like shape of cv curves. This shape is typical for the insertion of ions into host materials that exhibit a pseudocapacitive type of energy storage mechanism^30^. In such materials, redox couple activity might not be well-defined. It is believed that in the studied case potassium ions intercalate to the SnS_x_ structure according to the mechanism:

6 $${\text{SnS}}_{{\text{x}}} + {\text{yK}}^{ + } + {\text{ye}}^{ - } \to {\text{K}}_{{\text{y}}} {\text{Sn}}^{{({4} - {\text{y}})}} {\text{S}}_{{\text{x}}} \quad {\text{for}}\quad 0 < {\text{y}} < {2}$$

when x = 2 and SnS_x_ material has an ordered structure.

Only SnS_x_@C_s material fulfils these requirements. The reaction given in Eq. (6) results from the fact that the reduction of stannic to stannous ions is accompanied by the insertion of positively charged K^+^ and the electrode material, as a whole, remains electrically neutral. In the case of SnS_x_@C_sp, the amorphous SnS_2_ is not willing to react with potassium ions. Moreover, the crystal structure of SnS does not either react with K^+^ to form Sn as presented in Eq. (3). This is the reason why SnS_x_@C_s electrode material exhibits more stable electrochemical performance in comparison with SnS_x_@C_sp material for the 2nd and the 5th cycle, see Fig. 4.

**Fig. 5:**
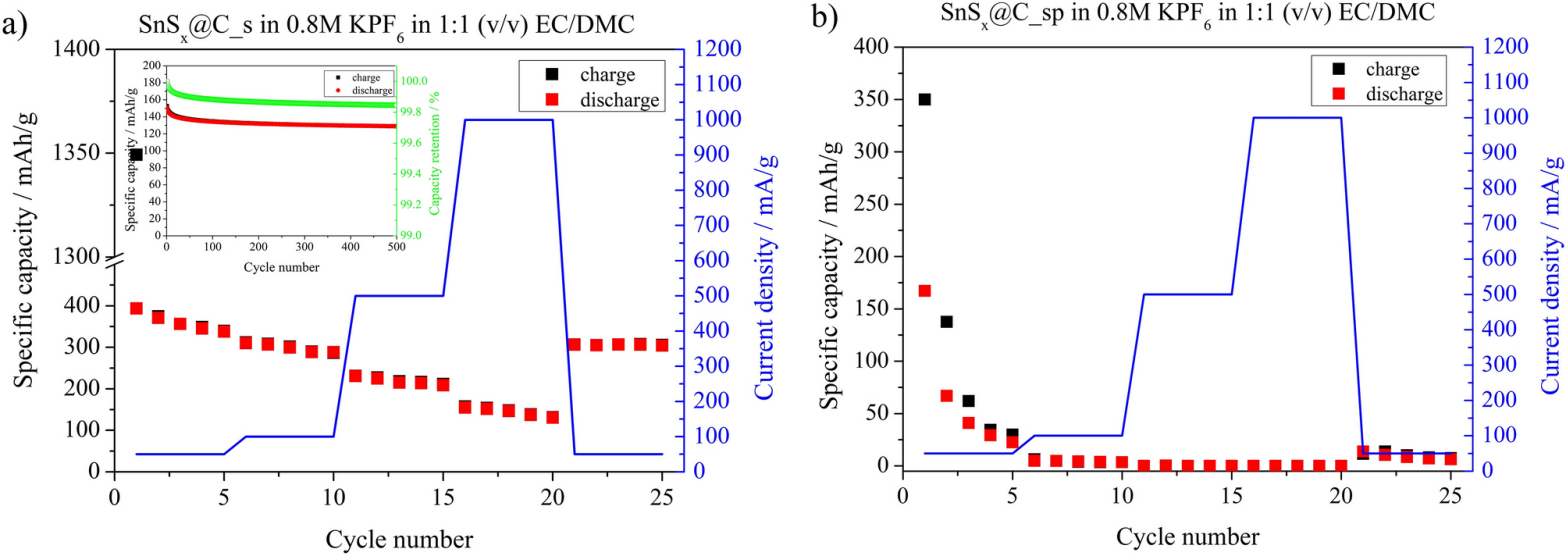
Rate capability of the (**a**) SnS_x_@C_s, and (**b**) SnS_x_@C_sp electrode materials at different current density in the potential range from 0.01 V to 2.0 V vs. K/K^+^. Inset in (**a**). Long-term cycles recorded at 1000 mA/g.

The rate capability of both electrode materials at current density changing from 50 mA/g to 1000 mA/g is presented in Fig. 5. The specific charge capacity for *j* = 50 mA/g after the 5th cycle was ~ 338 mAh/g for SnS_x_@C_s and ~ 30 mAh/g for SnS_x_@C_sp. After increasing current density to 100 mA/g, 500 mA/g, and 1000 mA/g, the capacity value for SnS_x_@C_s and SnS_x_@C_sp was 288 mAh/g, 208 mAh/g and 130 mAh/g for the 5th cycle, respectively. When the current density was decreased to 50 mA/g, the specific charge capacity of 304 mAh/g was reached, showing a capacity fade of 10%. Moreover, to evaluate long-term cyclability at high current density, 500 cycles at 1000 mA/g were performed, see inset in Fig. 5a. The initial specific charge capacity was 152 mAh/g and the final value was 128 mAh/g, giving a capacity retention of 84% after the 500th cycle. On the other hand, when current density was increased up to 1000 mA/g for the SnS_x_@C_sp electrode material, the specific capacity reached the level of ~ 1 mAh/g (see, Fig. 5b). Furthermore, even when the current density was back to 50 mA/g, the specific capacity was equal to ~ 10 mAh/g. The results indicate that pyrolysis has an undesirable effect on the electrochemical performance of the electrode material due to the vanishing of the crystallinity of SnS_2_. The presence of pyrolysed carbon, while intended to enhance certain properties, can inadvertently hinder ion transport and compromise overall battery performance. Thus, the SnS_x_@C_sp electrode material was not investigated in detail further. However, the comparison of charge/discharge curves of SnS_x_@C_s (Fig. S4a) and SnS_x_@C_sp (Fig. S4b) at different current densities are shown in supplementary information.

The results of the specific capacity for the studied tin sulfide-based materials compared with the literate date are given in Table 1.

**Table 1:** Comparison of tin-based anode materials in potassium-ion batteries.

Material	Specific capacity (mAh/g)	Current density (mA/g)	Reference
SnS_2_@N-rGO	645.2	50	^31^
	402	1000	^31^
Sn_4_P_3_/C	384.8	50	^32^
	221.9	1000	^32^
SnS/WS_2_@C	619	200	^33^
SnS_2_	36	100	^34^
N, S-C/SnS_2_	614.8	100	^35^
	104.5	2000	^35^
a-SnS@pCNFs	300	1000	^36^
SnS_x_@C_s	304	50	In this work
	128	1000	In this work

**Fig. 6:**
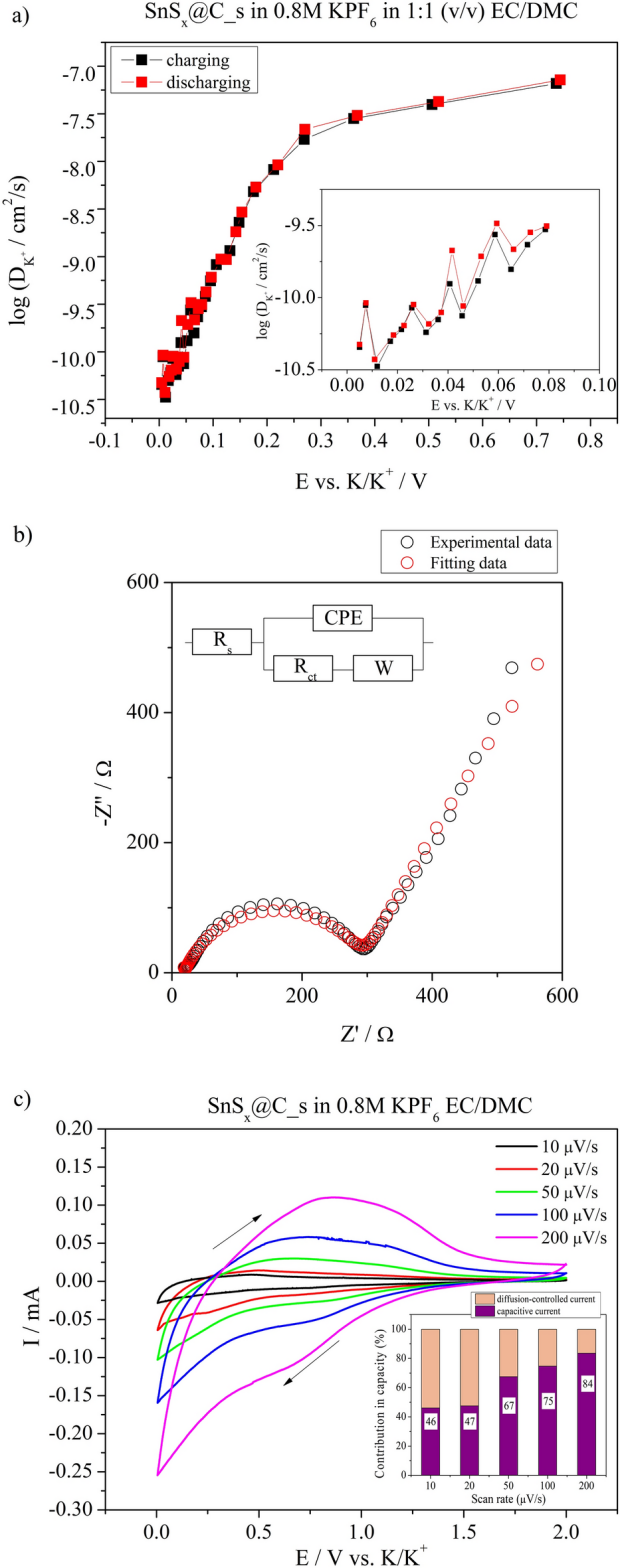
(**a**) Chemical diffusion coefficient (log D) as a function of the for SnS_x_@C_s electrode’s potential analyzed by GITT; (**b**) Nyquist plot for SnS_x_@C_s electrode material with experimental, fitted data, and electric equivalent circuit and (**c**) Cyclic voltammetry curves at different sweep rates in the potential range from 0.005 to 2.0 V vs. K/K^+^. Inset: Comparison of charge storage mechanism contribution for SnS_x_@C_s at different sweep rates.

To study the transport of potassium ions into the SnS_x_@C_s and SnS_x_@C_sp, the diffusion coefficient of K^+^ (${D}_{{K}^{+}}$) was measured with the utilization of galvanostatic intermittent titration technique (GITT)^37^, which is considered one of the most reliable methods in this field^38^. In the case of SnS_x_@C_s, it is possible to determine only the apparent diffusion coefficient because it is not a pure material but a composite consisting of carbonaceous and inorganic parts. Taking into account that diffusion within the bulk electrode is the limiting step compared to potassium ion transfer from the electrolyte to the surface, a simplified formula was used to calculate the chemical diffusion coefficient^39^:

7 $$D = \frac{{4 \cdot l^{2} }}{{\pi \cdot \varDelta t_{p} }}\left( {\frac{{\varDelta E_{s} }}{{\varDelta E_{t} }}} \right)^{2} \quad {\text{at t}} \ll \tau$$

where l is the diffusion length (the film thickness); Δ*t*_*p*_—time of the galvanostatic pulse duration; Δ*E*_*s*_—the change of a steady-state (equilibrium) voltage at the end of two sequential open-circuit relaxation periods; Δ*E*_*t*_—the total change in the cell voltage during the current pulse. This approach is reliably used as small currents and short time intervals are applied ($t \ll \frac{{l^{2} }}{D}$). Thus, semi-infinite diffusion of potassium ions takes place in the bulk of the electrode material. The calculated values of ${D}_{{K}^{+}}$ at different potential values were plotted in Fig. 6a for SnS_x_@C_s and in Fig. S5 for SnS_x_@C_sp. It is seen that as the potential decreased, the value of the diffusion coefficient also decreased. Such a phenomenon is related to narrowing the passageway of the ions into the host material during the insertion process^40^. The obtained values change from 7.17·10^−8^ to 4.7·10^−11^ cm^2^/s at 0.005 V for SnS_x_@C_s. In the case of SnS_x_@C_sp, the calculated values of the diffusion coefficient were 6 orders of magnitude lower. It is noteworthy to mention that the values of ${D}_{{K}^{+}}$ at a given potential were similar regarding both the reduction and oxidation processes. Such low values are typical for ion diffusion of potassium ions in solid materials^41,42^. For the SnS_x_@C_s electrode material electrochemical impedance spectroscopy was investigated before charge/discharge tests at E = 3.0 V, and the results are presented in Fig. 6b. The experimental Nyquist plot was modelled based on a modified Randles circuit (see inset in Fig. 6b). The impedance spectrum consists of one semicircle and a straight-sloping line. The semicircle is attributed to the charge transfer process at the electrolyte/electrode interface while the straight line is a Warburg impedance that represents potassium ion diffusion within the bulk material. The diffusion coefficient of potassium ions can be calculated from the Warburg diffusion coefficient (σ_w_), according to Eq. 8:

8 $$D_{{K^{+} }} = \left( {\frac{R \cdot T}{{A \cdot n^{2} \cdot F^{2} \cdot c \cdot \sigma_{W} }}} \right)^{2}$$

where R is the gas constant, T is the room temperature, A is the surface area of the electrode, n is the number of electrons per molecule in the reaction, F is the Faraday constant, and C is the concentration of K ions in the electrode. The σ_w_ was calculated from the slope of line fitting of Z’ versus ω^−1/2^^43^. The calculated ${D}_{{K}^{+}}$ was 3.6·10^−8^ cm^2^/s, and it was in the order of magnitude similar to the values obtained from GITT measurements.

According to the reaction given in Eqs. (1) and (2), it is expected that the potassium ion storage mechanism is related to the alloy reaction. However, taking into account the presence of the carbon matrix and the shape of cv curves for both electrode materials, it is assumed that the process related to charge storage is more complex, and may follow by the reaction given in Eq. (6). It is known that in the case of carbon-based composite materials, the reversible capacity may be coupled with the capacitive behaviour of the material^44^. Thus, the investigation regarding the mechanisms of energy storage was performed. To distinguish the nature of energy storage mechanisms, the method proposed by Conway was applied^45^ that enables distinguishing if the process involves a surface or diffusion-controlled mechanism, see Fig. 6c. In most cases, there is no simple division between these mechanisms. According to Eq. (9):

9 $${\text{i}}({\text{V}}) = {\text{k}}_{{1}} {\text{v}} + {\text{k}}_{{2}} {\text{v}}^{{{1}/{2}}}$$

the total current response is divided into surface (k_1_v) and diffusion-controlled (k_2_v^1/2^) process contribution. Thus, by rearranging Eq. (8) into $\frac{i\left(V\right)}{{v}^{1/2}}={k}_{1}{v}^{1/2}+{k}_{2}$, it is possible to quantify, at a specified potential, the current contribution to the individual energy storage mechanisms. The results of the analysis are shown as a bar chart in the inset in Fig. 6c, indicating that for 10 and 20 µV/s the charge storage mechanism is almost equally controlled by both capacitive and diffusion processes. However, with the sweep rate increase, the contribution of capacitive current increases, which is coherent with the GITT measurements and confirms that the diffusion of potassium ions is a slow process, affecting the faradaic reaction and diffusion-controlled mechanism, see Fig. 6c. Thus, the surface mechanism in the SnS_x_@C_s electrode material has a major contribution to the storage mechanism for sweep rates higher than 50 µV/s. Moreover, the presence of a carbon matrix, that covers the SnS_x_ particles, determines the energy storage mechanism to be of a capacitive nature, as confirmed by Goikolea et al. for carbon materials^46^, but does not affect the total value of the specific capacity of the electrode material.

**Fig. 7:**
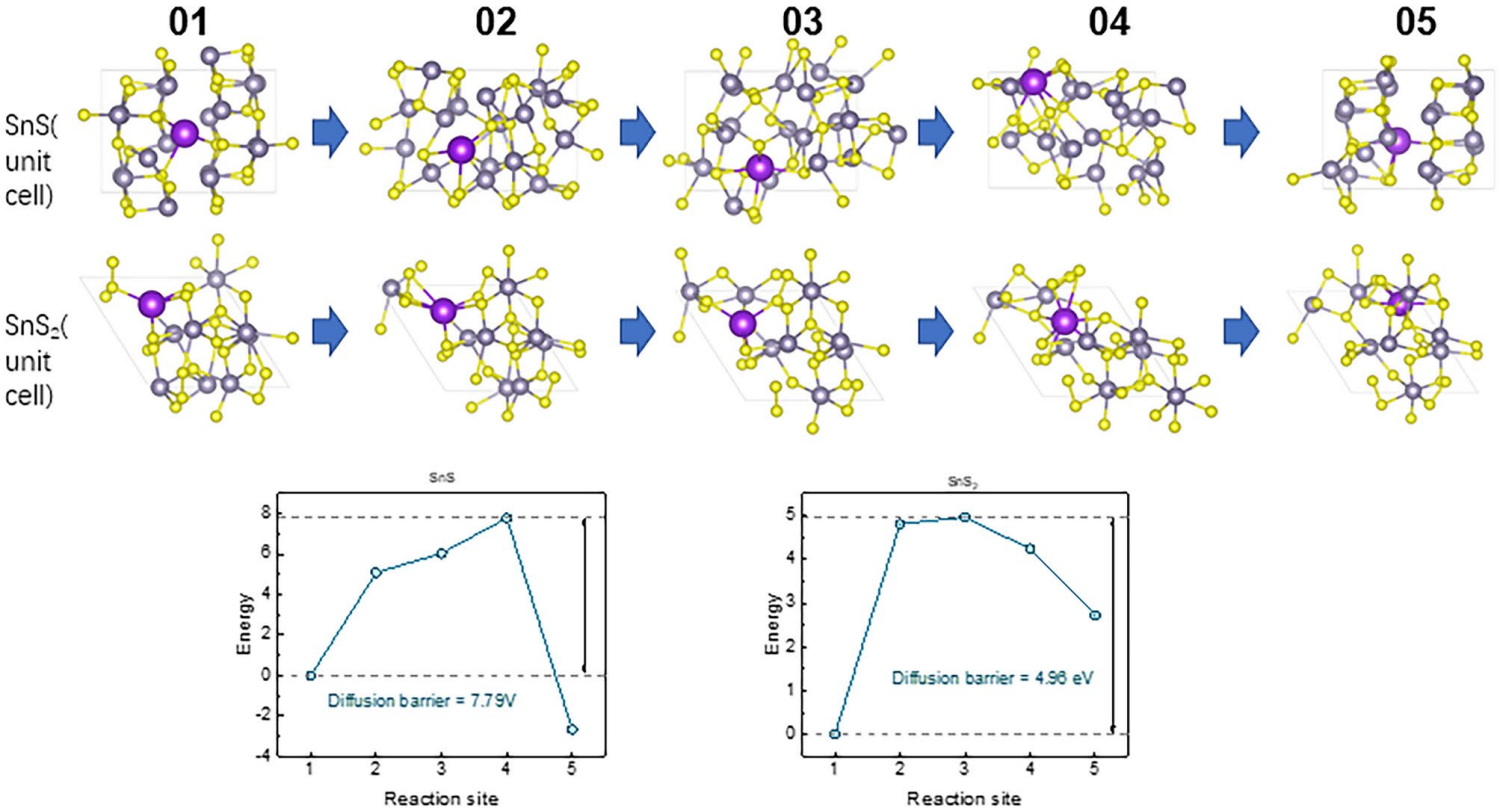
The schematic diagram of the initial (01) and final (05) stage of K^+^ position in the SnS and SnS_2_ crystals.

To investigate the reason for such poor electrochemical behaviour of SnS_x_@C_sp in comparison with the SnS_x_@C_s electrode material, the DFT calculations were performed. First, the migration energy barrier of K^+^ in orthorhombic SnS and hexagonal SnS_2_ crystals was calculated. The principle of the Climbing Image-Nudged Elastic Band (CI-NEB) method, involving the insertion of a series of structures between the reactants and the products, totalling three points, was utilized. Instead of optimizing each point in isolation, the optimization process improved a function where all points moved together at each step. In Fig. 7, 01 and 05 represent the initial and final state structure diagrams, respectively, while 02 to 04 depict the intermediate states.

**Fig. 8:**
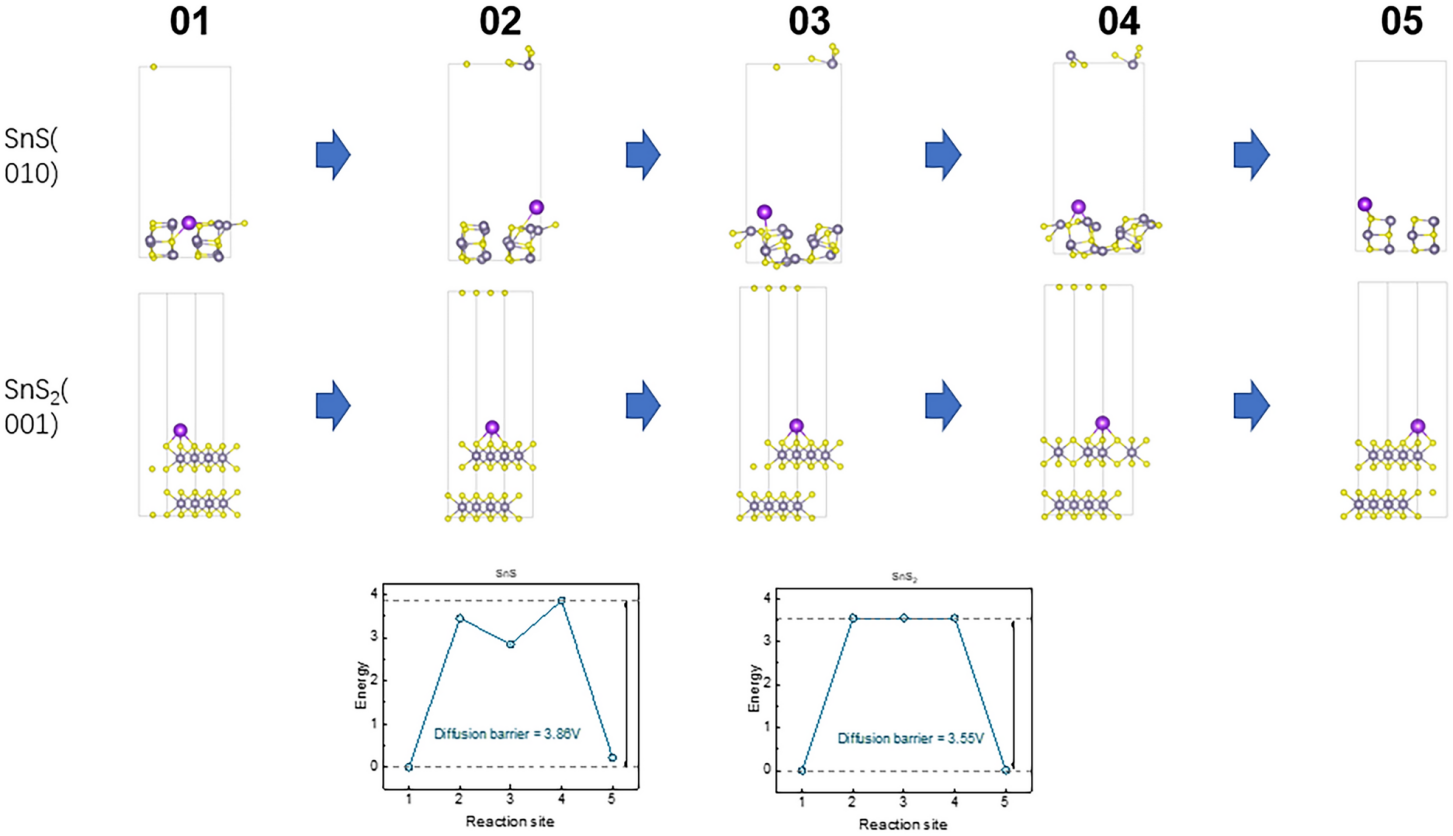
The schematic diagram of the initial (01) and final (05) of K^+^ position on the surface of SnS (010) plane and (001) SnS_2_ plane.

The energy barrier diagrams in Fig. 7 show the energy changes for the structure at each state. It is observed that the diffusion energy barrier of K^+^ in SnS is significantly higher (7.79 eV) than in SnS_2_ (4.96 eV), indicating that K^+^ diffusion in SnS is more challenging which is in agreement with GITT results. This is primarily attributed to stronger atomic-level interactions between K⁺ and the SnS lattice. In SnS, the tightly packed lattice structure forces K^+^ to navigate migration paths through smaller gaps, constrained by the surrounding Sn and S atoms. These constraints significantly hinder K^+^ diffusion. In contrast, SnS₂ features a layered structure with larger interlayer spacing, providing more spacious diffusion channels. This structural arrangement allows K^+^ ions to move freely within the interlayer regions, encountering significantly less hindrance. It confirmed that orthorhombic SnS was not the best choice as an electrode material for potassium ions insertion in comparison with SnS_2_. Additionally, the energy barrier for potassium ion diffusion on the surface of the SnS (010) plane and the SnS_2_ (001) plane were also modelled. It turned out that the diffusion energy barrier of K^+^ in SnS was also higher than in SnS_2_, see Fig. 8, but the difference was only 0.31 eV. It evidenced that the diffusion of potassium ions at the electrolyte/electrode interphase is also more difficult in the case of SnS than in SnS_2_. Moreover, this observation reinforces our previous findings, highlighting the critical role of crystal plane orientation in facilitating the ion insertion process into the host material. Specifically, the confirmation of this relationship underscores the significance of crystallographic factors in governing the kinetics and mechanism of ion insertion and extraction within the electrode material^47^.

It is noteworthy that computational analyses of tin-derived materials, such as monolayer SnS_2_^48^, SnSe_2_^49^, as anode materials for potassium- or sodium-ion batteries, confirm that the utilization of DFT calculations aligns well with experimental data. For instance, studies have demonstrated that the layered structure of SnS_2_ facilitates ion diffusion due to its larger interlayer spacing, which is consistent with the presented findings. Computational insights into similar materials have shown that the intrinsic crystal structure and atomic-level interactions play a pivotal role in determining ion storage and transport properties.

## Conclusions

Two electrode materials based on the SnS_x_@C system were synthesized. The first SnS_x_@C_s was obtained by the solvothermal reaction, while another SnS_x_@C_sp was a result of pyrolysis of the former one. It was evidenced that the synthesis method affected the material composition as well as the crystallographic structure. The hexagonal SnS_2_ crystals were identified in SnS_x_@C_s while the orthorhombic phase of SnS was present in SnS_x_@C_sp. XPS analysis confirmed the presence of only SnS_2_ in SnS_x_@C_s, although pyrolysis of SnS_x_@C_s led to additional formation of SnS in the material. Pyrolysis was a crucial factor in determining the structure of electrode material and thus affected its electrochemical performance. It turned out that the SnS_x_@C_s electrode material exhibited much better electrochemical performance in comparison with the SnS_x_@C_sp material. The former showed a specific capacity of 128 mAh/g at* j* = 1000 mA/g with a capacity retention of 84% after 500 cycles. The Raman spectroscopy investigation confirmed the presence of carbon matrix consisting of disordered and ordered graphitic species. The calculated particle size of the crystalline carbon domains were of the same order confirming that the synthesis method did not influence the structure of carbon significantly. The DFT approach showed that diffusion of potassium ions on the surface of materials is more difficult for SnS (SnS_x_@C_sp) than for SnS_2_ (SnS_x_@C_s). Additionally, the diffusion of potassium ions into the host material is much easier for the solvothermally obtained material showing a diffusion energy barrier of 4.96 eV in comparison with 7.79 eV for the second material. The collected results reveal that hexagonal SnS_2_ might be suitable as a negative electrode for potassium ion batteries much better than orthorhombic SnS.

## Supplementary Information

Supplementary Information 1.

## Data Availability

The datasets generated and/or analyzed during the current study are available in the BRIDGE OF KNOWLEDGE repository (https://mostwiedzy.pl/pl/open-research-data/xrd-database-for-sns-based-electrode-material,1125024837113798-0, https://doi.org/10.34808/0xh1-ts89).

## References

[1] IEA. Renewables 2021. *Int. Energy Agency Publ. Int.* **167** (2021).

[2] Wang, C., Yang, C. & Zheng, Z. Toward practical high-energy and high-power lithium battery anodes: Present and future. *Adv. Sci.* **9**, 1–16 (2022). 10.1002/advs.202105213 PMC8948585 35098702

[3] Mojtaba, M., Abbas, Q., Hunt, M. R. C., Galeyeva, A. & Raza, R. Na-Ion batteries. *Encycl. Smart Mater.* **2**, 135–147 (2022).

[4] Min, X. et al. Potassium-ion batteries: Outlook on present and future technologies. *Energy Environ. Sci.* **14**, 2186–2243 (2021).

[5] Zhang, J. et al. Model-based design of stable electrolytes for potassium ion batteries. *ACS Energy Lett.* **5**, 3124–3131 (2020).

[6] Zheng, J. et al. Recent advances in potassium-ion batteries: From material design to electrolyte engineering. *Adv. Mater. Technol.* **8**, 1–26 (2023).

[7] Hundekar, P. et al. In situ healing of dendrites in a potassium metal battery. *Proc. Natl. Acad. Sci. U. S. A.* **117**, 5588–5594 (2020). 32123085 10.1073/pnas.1915470117 PMC7084065

[8] Xu, Y. *et al.* 2023 Roadmap for potassium-ion batteries. *J. Phys.: Energy OPEN ACCESS* (2023).

[9] Inamuddin, Boddula, R. & Asiri, A. M. *Potassium-Ion Batteries* (Wiley, 2020). 10.1002/9781119663287

[10] Qu, B. et al. Layered SnS_2_-reduced graphene oxide composite—A high-capacity, high-rate, and long-cycle life sodium-ion battery anode material. *Adv. Mater.* **26**, 3854–3859 (2014). 24677348 10.1002/adma.201306314

[11] Abutbul, R. E. et al. Crystal structure of a large cubic tin monosulfide polymorph: An unraveled puzzle. *CrystEngComm* **18**, 5188–5194 (2016).

[12] Kubota, K., Dahbi, M., Hosaka, T., Kumakura, S. & Komaba, S. Towards K-ion and Na-ion batteries as “beyond Li-ion”. *Chem. Rec.* **18**, 459–479 (2018). 29442429 10.1002/tcr.201700057

[13] Li, D. et al. Surface-confined SnS_2_@C@rGO as high-performance anode materials for sodium- and potassium-ion batteries. *ChemSusChem* **12**, 2689–2700 (2019). 30997950 10.1002/cssc.201900719

[14] Sun, Q., Li, D., Dai, L., Liang, Z. & Ci, L. Structural engineering of SnS_2_ encapsulated in carbon nanoboxes for high-performance sodium/potassium-ion batteries anodes. *Small* **16**, 1–10 (2020). 10.1002/smll.202005023 33079488

[15] Liu, J., Yu, X., Bao, J., Sun, C. F. & Li, Y. Carbon supported tin sulfide anodes for potassium-ion batteries. *J. Phys. Chem. Solids* **153** (2021).

[16] Wang, L., Bao, J., Liu, Q. & Sun, C. F. Concentrated electrolytes unlock the full energy potential of potassium-sulfur battery chemistry. *Energy Storage Mater.* **18**, 470–475 (2019).

[17] Nowak, A. P. et al. Tin oxide encapsulated into pyrolyzed chitosan as a negative electrode for lithium ion batteries. *Materials (Basel).* **14**, 1156–1167 (2021). 33804496 10.3390/ma14051156 PMC7957769

[18] Song, J. *et al.* SnS/C nanostructures endowed by low-temperature in-situ carbothermal reduction of sustainable lignin for stable lithium- and sodium-ion storage. *Chem. Eng. Sci.* **300** (2024).

[19] Ferrari, A. C. & Robertson, J. Interpretation of Raman spectra of disordered and amorphous carbon. *Phys. Rev. B* **61**, 14095–14107 (2000).

[20] Sadezky, A., Muckenhuber, H., Grothe, H., Niessner, R. & Pöschl, U. Raman microspectroscopy of soot and related carbonaceous materials: Spectral analysis and structural information. *Carbon N. Y.* **43**, 1731–1742 (2005).

[21] Tuinstra, F. & Koenig, J. L. Raman spectrum of graphite. *J. Chem. Phys.* **1126**, 1126–1130 (1970).

[22] Nowak, A. P. *et al.* Lignin-based carbon fibers for renewable and multifunctional lithium-ion battery electrodes. *Holzforschung* **72** (2018).

[23] Li, L. et al. Single-atom Ce targeted regulation SnS/SnS_2_ heterojunction for sensitive and stable room-temperature ppb-level gas sensor. *Chem. Eng. J.* **472**, 144796 (2023).

[24] Liu, K. et al. Simple construction and reversible sequential evolution mechanism of nitrogen-doped mesoporous carbon/SnS_2_ nanosheets in lithium-ion batteries. *Appl. Surf. Sci.* **618**, 156673 (2023).

[25] Witkowski, M., Starowicz, Z. & Zi, A. The atomic layer deposition ( ALD ) synthesis of copper-tin sul fi de thin fi lms using low- cost precursors. *Nanotechnology* **33**, 505603 (2022). 10.1088/1361-6528/ac9065 36075187

[26] Szkoda, M., Zarach, Z., Trzciński, K., Trykowski, G. & Nowak, A. P. An easy and ecological method of obtaining hydrated and non-crystalline WO3-x for application in supercapacitors. *Materials (Basel).* **13** (2020). 10.3390/ma13081925 PMC7215928 32325884

[27] Oliveira, J. R., Martins, M. C. L., Mafra, L. & Gomes, P. Synthesis of an O-alkynyl-chitosan and its chemoselective conjugation with a PEG-like amino-azide through click chemistry. *Carbohydr. Polym.* **87**, 240–249 (2012). 34662957 10.1016/j.carbpol.2011.07.043

[28] Szewczyk, I. et al. Electrochemical denitrification and oxidative dehydrogenation of ethylbenzene over N-doped mesoporous carbon: Atomic level understanding of catalytic activity by 15N NMR spectroscopy. *Chem. Mater.* **32**, 7263–7273 (2020).

[29] Nowak, A. P. *et al.* A negative effect of carbon phase on specific capacity of electrode material consisted of nanosized bismuth vanadate embedded in carbonaceous matrix. *Synth. Met.* **257** (2019).

[30] Gogotsi, Y. & Penner, R. M. Energy storage in nanomaterials—Capacitive, pseudocapacitive, or battery-like?. *ACS Nano* **12**, 2081–2083 (2018). 29580061 10.1021/acsnano.8b01914

[31] Sheng, C. et al. SnS_2_/N-doped graphene as a superior stability anode for potassium-ion batteries by inhibiting “Shuttle effect”. *Batter. Supercaps* **3**, 56–59 (2020).

[32] Huang, B., Pan, Z., Su, X. & An, L. Tin-based materials as versatile anodes for alkali (earth)-ion batteries. *J. Power Sources* **395**, 41–59 (2018).

[33] Zeng, X. et al. Hierarchical nanocomposite of hollow N-doped carbon spheres decorated with ultrathin WS2 nanosheets for high-performance lithium-ion battery anode. *ACS Appl. Mater. Interfaces* **8**, 18841–18848 (2016). 27381381 10.1021/acsami.6b04770

[34] Li, C. *et al.* Constructing hollow microcubes SnS_2_ as negative electrode for sodium-ion and potassium-ion batteries. *Chem. A Eur. J.* **30**, e202304296 (1 of 11) (2024). 10.1002/chem.202304296 38380537

[35] Li, W., Yang, Z., Zuo, J., Wang, J. & Li, X. Emerging carbon-based flexible anodes for potassium-ion batteries: Progress and opportunities. *Front. Chem.* **10**, 1–16 (2022). 10.3389/fchem.2022.1002540 PMC9493046 36157035

[36] Li, R. et al. Embedding amorphous SnS in electrospun porous carbon nanofibers for efficient potassium storage with ultralong cycle life. *Compos. Part B Eng.* **243**, 110132 (2022).

[37] Weppner, W. & Huggins, R. A. Determination of the kinetic parameters of mixed-conducting electrodes and application to the system Li3Sb. *J. Electrochem. Soc.* **124**, 1569–1578 (1977).

[38] Nickol, A. et al. GITT analysis of lithium insertion cathodes for determining the lithium diffusion coefficient at low temperature: Challenges and pitfalls. *J. Electrochem. Soc.* **167**, 090546 (2020).

[39] Wen, C. J., Boukamp, B. A. & Huggins, R. A. Thermodynamic and mass transport properties of “LiIn”. *Mater. Res. Bull.* **15**, 1225–1234 (1980).

[40] NuLi, Y., Yang, J. & Jiang, Z. Intercalation of lithium ions into bulk and powder highly oriented pyrolytic graphite. *J. Phys. Chem. Solids* **67**, 882–886 (2006).

[41] Kahlert, H., Retter, U., Lohse, H., Siegler, K. & Scholz, F. On the determination of the diffusion coefficients of electrons and of potassium ions in copper(II) hexacyanoferrate(II) composite electrodes. *J. Phys. Chem. B* **102**, 8757–8765 (1998).

[42] He, J. *et al.* Rational construction of advanced potassium ion diffusion and storage matrix. *Adv. Funct. Mater.* **31** (2021).

[43] Feng, M. et al. Manganese oxide electrode with excellent electrochemical performance for sodium ion batteries by pre-intercalation of K and Na ions. *Sci. Rep.* **7**, 1–8 (2017). 28533510 10.1038/s41598-017-02028-0 PMC5440409

[44] Szkoda, M., Zarach, Z., Trzciński, K. & Nowak, A. P. An aqueous exfoliation of WO3 as a route for counterions fabrication—improved photocatalyticand capacitive properties of polyaniline/WO3 composite. *Materials (Basel).* **13**, 1–16 (2020). 10.3390/ma13245781 PMC7766862 33348911

[45] Liu, T.-C., Pell, W. G., Conway, B. E. & Roberson, S. L. Behavior of molybdenum nitrides as materials for electrochemical capacitors. *J. Electrochem. Soc.* **145**, 1882–1888 (1998).

[46] Goikolea, E. & Mysyk, R. *Nanotechnology in Electrochemical Capacitors*. *Emerging Nanotechnologies in Rechargeable Energy Storage Systems* (Elsevier Inc., 2017). 10.1016/B978-0-323-42977-1.00004-2

[47] Trzciński, K. et al. Controlling crystallites orientation and facet exposure for enhanced electrochemical properties of polycrystalline MoO_3_ films. *Sci. Rep.* **13**, 1–12 (2023). 37794143 10.1038/s41598-023-43800-9 PMC10550991

[48] Rehman, J., Fan, X. & Zheng, W. T. Computational insight of monolayer SnS_2_ as anode material for potassium ion batteries. *Appl. Surf. Sci.* **496**, 143625 (2019).

[49] Rehman, J. et al. First principles predictions of Na and K storage in layered SnSe_2_. *Appl. Surf. Sci.* **566**, 150522 (2021).

