# Supplementary material for: Experimental and computational analysis of SnSx encapsulated into carbonized chitosan as electrode material for potassium ion batteries
Source: Sci Rep. 2024 Dec 28;14:31212. doi: 10.1038/s41598-024-82588-0 (PMC11682189; doi:10.1038/s41598-024-82588-0)
Supplement: Supplementary file 1 — Supplementary Information 1. [file 41598_2024_82588_MOESM1_ESM.docx]

**Supplementary Material**

Experimental And Computational Analysis Of SnS_x_ Encapsulated Into Carbonized Chitosan As Electrode Material For Potassium Ion Batteries

Andrzej P. Nowak^1,2*^, Anna Rokicińska^3^_,_ Zhilong Wang^4,5^, Marta Prześniak-Welenc^2,6^, Zuzanna Zarach^1^, Kehao Tao^4,5^, Daria Roda^1^, Mariusz Szkoda^1,2^, Konrad Trzciński^1,2^, Jinjin Li^4&^, Piotr Kuśtrowski^3$^

^1^Faculty of Chemistry, Gdańsk University of Technology, Narutowicza 11/12, 80-233 Gdańsk, Poland.

^2^Advanced Materials Center, Gdańsk University of Technology, Narutowicza 11/12, 80-233 Gdańsk, Poland.

^3^Faculty of Chemistry, Jagiellonian University, Gronostajowa 2, Kraków 30-387, Poland

^4^National Key Laboratory of Advanced Micro and Nano Manufacture Technology, Shanghai Jiao Tong University, Shanghai, 200240, China

^5^Department of Micro/Nano Electronics, School of Electronic Information and Electrical Engineering, Shanghai Jiao Tong University, Shanghai, 200240, China

^6^Institute of Nanotechnology and Materials Engineering, Gdańsk University of Technology, Narutowicza 11/12, 80-233 Gdańsk

* andnowak@pg.edu.pl

& [lijinjin@sjtu.edu.cn](about:blank)

$ piotr.kustrowski@uj.edu.pl

**SEM/EDX**

SnS_x_@C_s

| 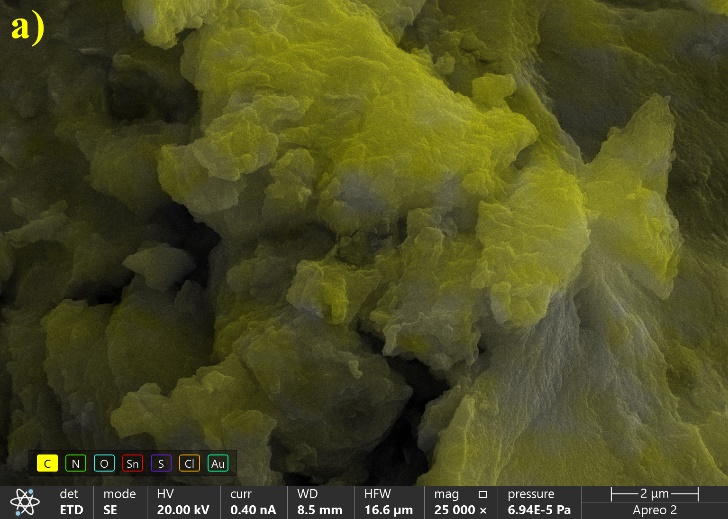 | 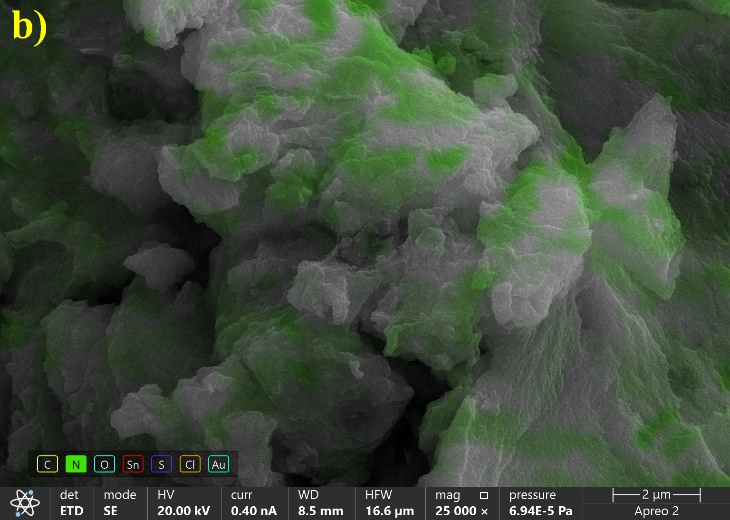 |
| --- | --- |
| 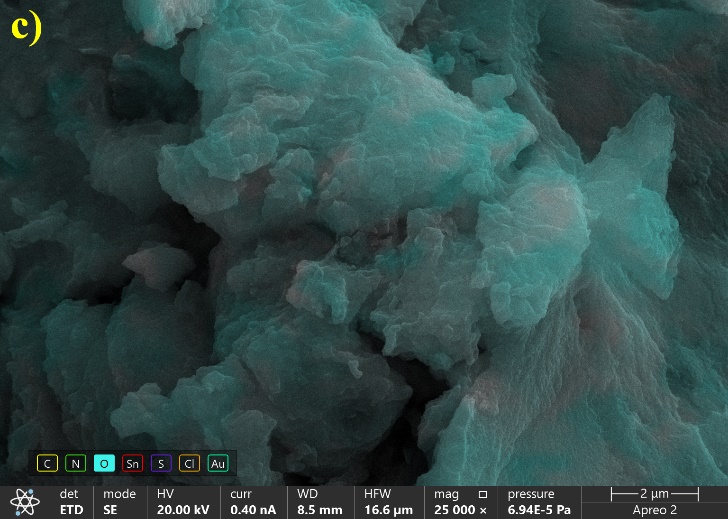 | 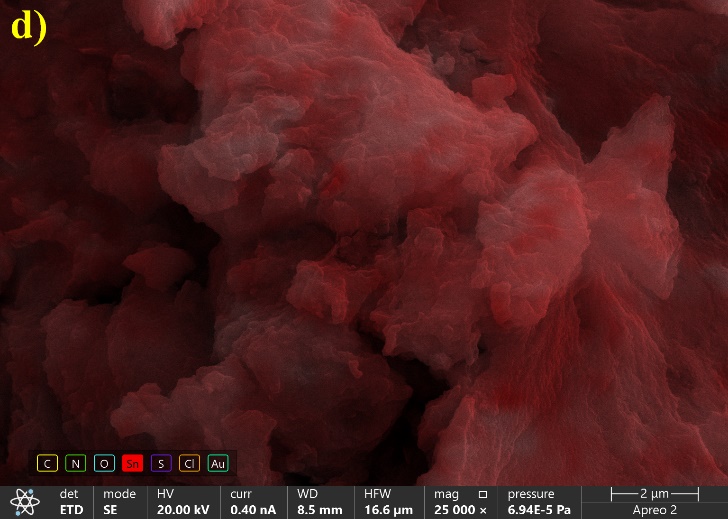 |
| 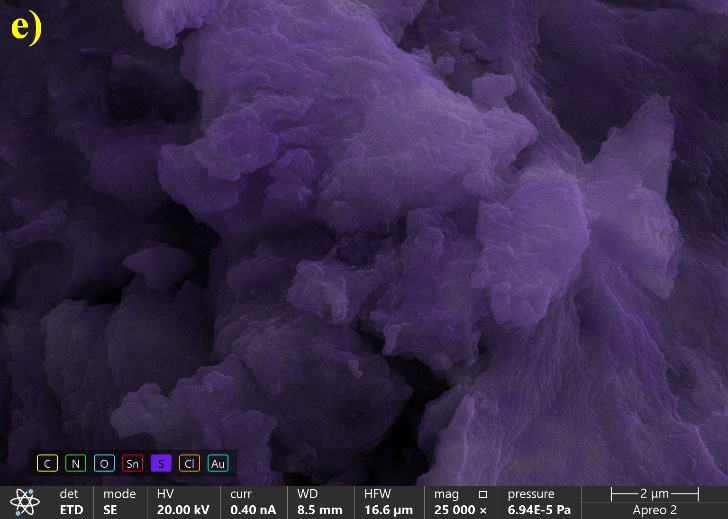 | 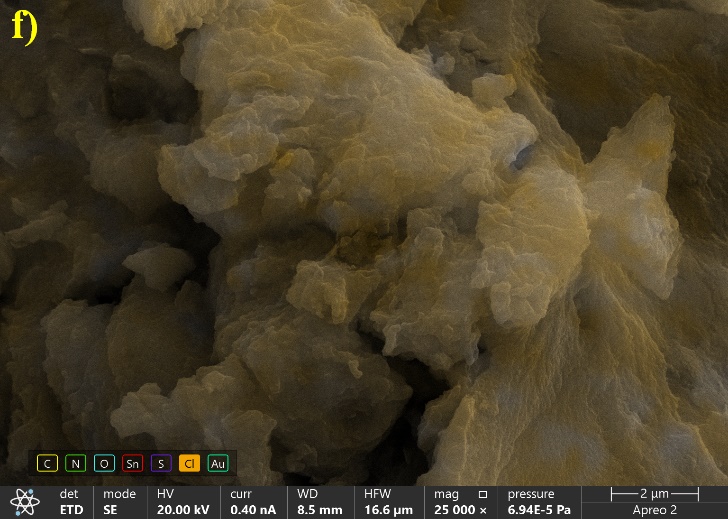 |
| **Figure S1** SEM images with EDX mapping of C (a), N (b), O (c), Sn (d), S (e), and Cl (f) for SnS_x_@C_s composite material. | |

**Table S1** Chemical composition of the SnS_x_@C_s material based on the EDX measurements.

| **Element** | **Atomic %** | **Atomic % Error** | **Weight %** | **Weight % Error** | **Net Counts** |
| --- | --- | --- | --- | --- | --- |
| Cl | 3.3 | 0.1 | 5.2 | 0.1 | 16 957 |
| C | 31.1 | 0.4 | 16.4 | 0.2 | 10 106 |
| N | 10.4 | 0.7 | 6.4 | 0.4 | 1 849 |
| O | 38.6 | 0.6 | 27.1 | 0.4 | 10 429 |
| S | 10.9 | 0.1 | 15.3 | 0.1 | 66 671 |
| Sn | 5.7 | 0.1 | 29.7 | 0.3 | 48 135 |

SnS_x_@C_sp

| 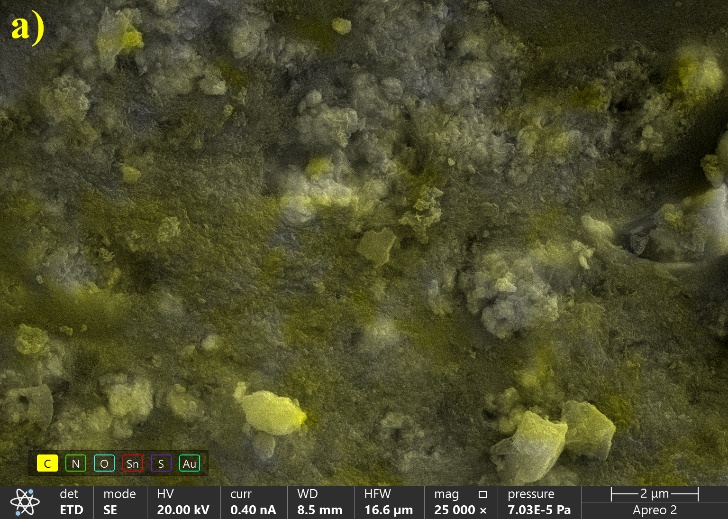 | 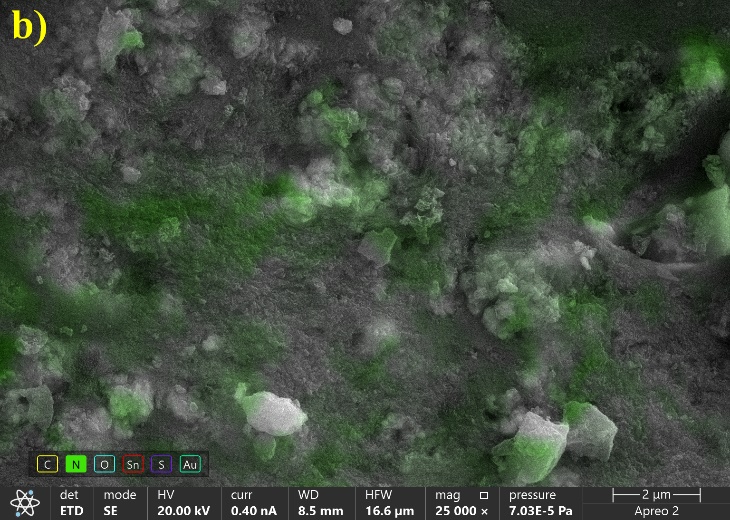 |
| --- | --- |
| 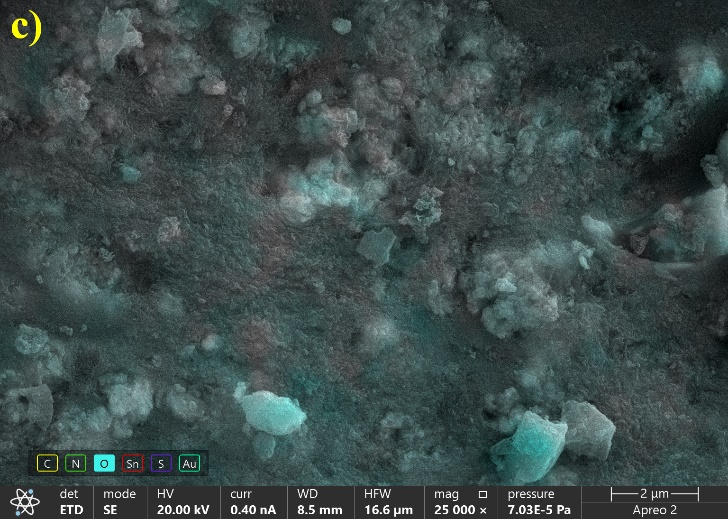 | 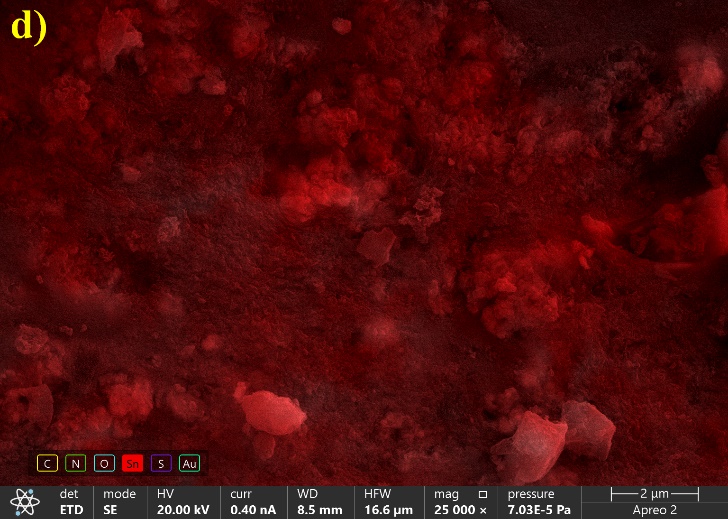 |
| 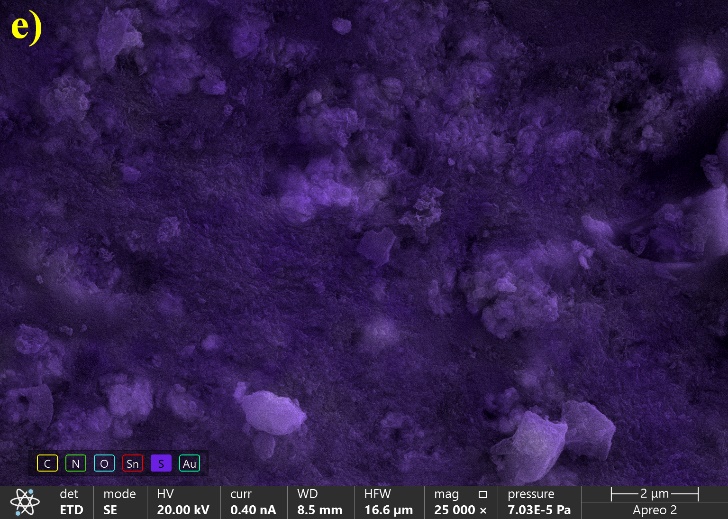 |  |
| **Figure S2** SEM images with EDX mapping of C (a), N (b), O (c), Sn (d), and S (e) for SnS_x_@C_s composite material. | |

**Table S2** Chemical composition of the SnS_x_@C_sp material based on the EDX measurements.

| **Element** | **Atomic %** | **Atomic % Error** | **Weight %** | **Weight % Error** | **Net Counts** |
| --- | --- | --- | --- | --- | --- |
| C | 45.1 | 0.4 | 22.6 | 0.2 | 21 616 |
| N | 12.2 | 1.1 | 7.1 | 0.6 | 2 476 |
| O | 23.5 | 0.6 | 15.7 | 0.4 | 6 455 |
| S | 11.2 | 0.1 | 15.0 | 0.1 | 76 842 |
| Sn | 8.0 | 0.1 | 39.6 | 0.4 | 79 105 |

**XPS**

**
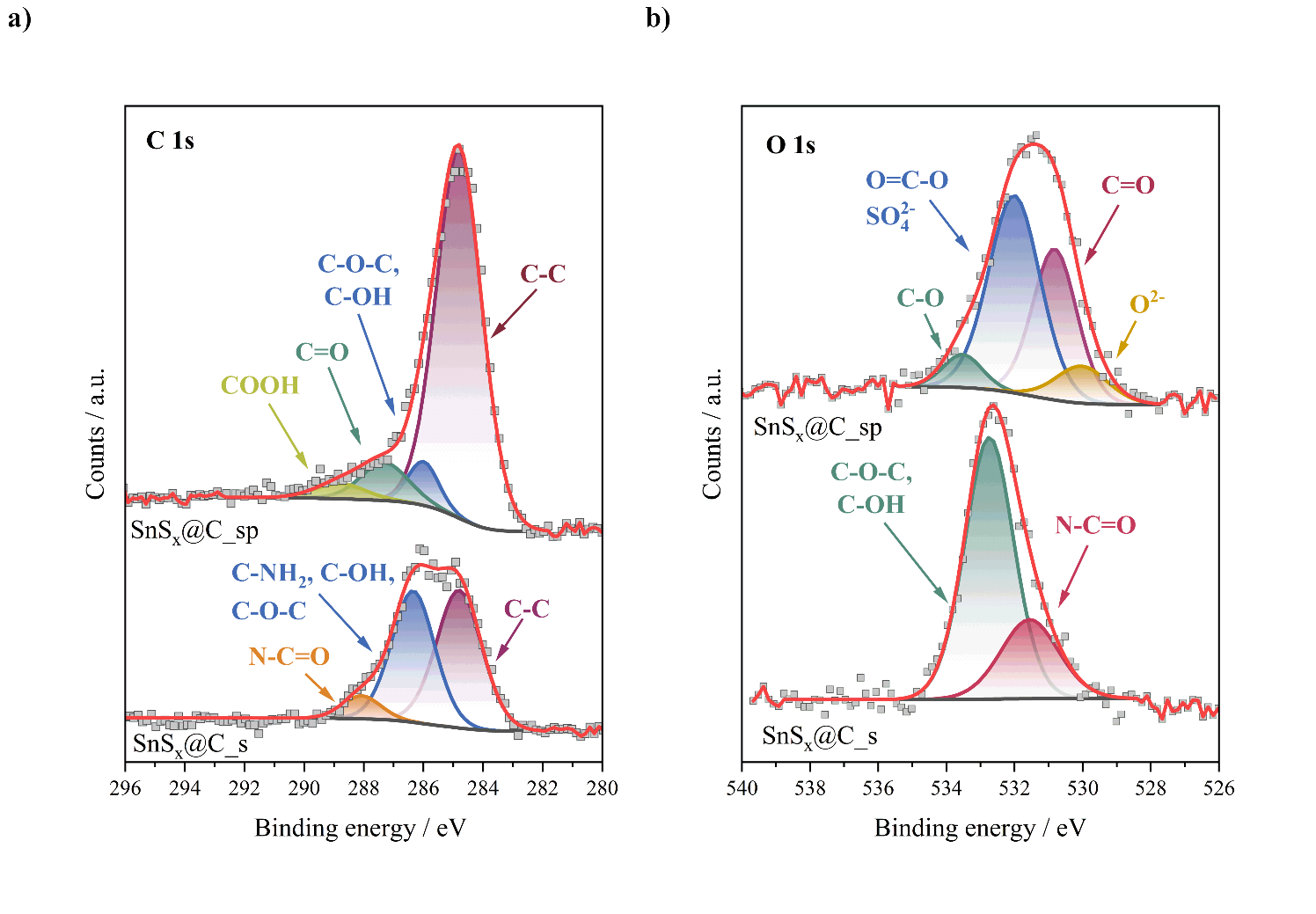
**

**Figure S3.** High resolution XPS spectra of A) C 1s and B) ) 1s for the SnS_x_@C_s and SnS_x_@C_sp electrode materials.

**Electrochemistry**

|  |  |
| --- | --- |
| **Figure S4** The charge-discharge curves for a) SnS_x_@C_s and b) SnS_x_@C_sp electrode materials. | |





**Figure S5** Chemical diffusion coefficient (log D) as a function of the SnS_x_@C_sp electrode’s potential analyzed by GITT.

**DFT**

**Table S3** The lattice parameter of tin sulfide-based materials taken to DFT calculations

|  | a (Å) | b (Å) | c (Å) | alpha (°) | beta (°) | gamma (°) |
| --- | --- | --- | --- | --- | --- | --- |
| SnS (010) | 9.07 | 11.39 | 23.96 | 90.0 | 90.0 | 90.0 |
| SnS | 9.07 | 11.39 | 7.91 | 90.0 | 90.0 | 90.0 |
| SnS_2_ (001) | 7.35 | 7.35 | 29.2 | 90.0 | 90.0 | 120.0 |
| SnS_2_ | 8.71 | 8.49 | 9.22 | 90.0 | 90.0 | 120.0 |
